# JIS Definition Identified More Malaysian Adults with Metabolic Syndrome Compared to the NCEP-ATP III and IDF Criteria

**DOI:** 10.1155/2013/760963

**Published:** 2013-09-23

**Authors:** Anis Safura Ramli, Aqil Mohammad Daher, Mohamed Noor Khan Nor-Ashikin, Nafiza Mat-Nasir, Kien Keat Ng, Maizatullifah Miskan, Krishnapillai S. Ambigga, Farnaza Ariffin, Md Yasin Mazapuspavina, Suraya Abdul-Razak, Hasidah Abdul-Hamid, Fadhlina Abd-Majid, Najmin Abu-Bakar, Hapizah Nawawi, Khalid Yusoff

**Affiliations:** ^1^Primary Care Medicine Discipline, Faculty of Medicine, Universiti Teknologi MARA, 47000 Sungai Buloh, Selangor, Malaysia; ^2^Centre for Translational Research and Epidemiology (CenTRE), Faculty of Medicine, Universiti Teknologi MARA, 47000 Sungai Buloh, Selangor, Malaysia; ^3^Population Health & Preventive Medicine Discipline, Faculty of Medicine, Universiti Teknologi MARA, 47000 Sungai Buloh, Selangor, Malaysia; ^4^Physiology Discipline, Faculty of Medicine, Universiti Teknologi MARA, 47000 Sungai Buloh, Selangor, Malaysia; ^5^Centre for Pathology & Diagnostic Research Laboratory (CPDRL), Faculty of Medicine, Universiti Teknologi MARA, 47000 Sungai Buloh, Selangor, Malaysia; ^6^Cardiology Discipline, Faculty of Medicine, Universiti Teknologi MARA, 47000 Sungai Buloh, Selangor, Malaysia

## Abstract

Metabolic syndrome (MetS) is a steering force for the cardiovascular diseases epidemic in Asia. This study aimed to compare the prevalence of MetS in Malaysian adults using NCEP-ATP III, IDF, and JIS definitions, identify the demographic factors associated with MetS, and determine the level of agreement between these definitions. The analytic sample consisted of 8,836 adults aged ≥30 years recruited at baseline in 2007–2011 from the Cardiovascular Risk Prevention Study (CRisPS), an ongoing, prospective cohort study involving 18 urban and 22 rural communities in Malaysia. JIS definition gave the highest overall prevalence (43.4%) compared to NCEP-ATP III (26.5%) and IDF (37.4%), *P* < 0.001. Indians had significantly higher age-adjusted prevalence compared to other ethnic groups across all MetS definitions (30.1% by NCEP-ATP III, 50.8% by IDF, and 56.5% by JIS). The likelihood of having MetS amongst the rural and urban populations was similar across all definitions. A high level of agreement between the IDF and JIS was observed (Kappa index = 0.867), while there was a lower level of agreement between the IDF and NCEP-ATP III (Kappa index = 0.580). JIS definition identified more Malaysian adults with MetS and therefore should be recommended as the preferred diagnostic criterion.

## 1. Introduction

Metabolic syndrome (MetS), which is also known as “Syndrome X,” was first described by Reaven in his 1988 Banting Lecture [1]. It is characterized by clustering of cardiovascular risk factors, namely, central obesity, elevated blood pressure, elevated plasma glucose, and dyslipidaemia [2]. Insulin resistance and adipose tissue dysfunction are thought to be the primary mediators of MetS [3, 4]. 

Since it was first introduced, MetS has become the subject of paramount research interest and also a subject of ongoing debate with regard to its clinical value as a distinct diagnostic entity [5, 6]. Nevertheless, several diagnostic criteria for MetS continued to emerge over the last few decades. The first definition was introduced by the World Health Organization (WHO) in 1998 [7] followed by the National Cholesterol Education Program Expert Panel on Detection, Evaluation, and Treatment of High Blood Cholesterol in Adults (NCEP-ATP III) in 2001 [8]. Subsequently, the International Diabetes Federation (IDF) established a new worldwide definition for MetS in 2005 [9]. In contrast to the previous definitions, IDF made abdominal obesity a mandatory criterion using waist circumference (WC) cut-points based on gender and ethnicity [9]. In the same year, the NCEP-ATP III was modified to incorporate a lower fasting glucose threshold of ≥5.6 mmol/L and the same WC criteria as proposed by the IDF for Asians living in the United States [10]. However, the modified NCEP-ATP III maintained that the diagnosis of MetS should be made based on the presence of any 3 out of 5 risk factors. After several years of continuing debate and discussion, the various stakeholders finally agreed to harmonize the definition. A Joint Interim Statement (JIS) on MetS definition was issued in 2009 by the IDF Task Force on Epidemiology and Prevention; National Heart, Lung, and Blood Institute; American Heart Association; World Heart Federation; International Atherosclerosis Society; and International Association for the Study of Obesity [11]. The JIS committee agreed that the presence of any 3 out of 5 risk factors constitutes a diagnosis of MetS. Abdominal obesity is no longer a prerequisite for diagnosis, as more evidence is needed to relate WC with the risk of developing type 2 diabetes mellitus (T2DM) and cardiovascular diseases (CVD) [11]. Long-term prospective studies are also required to establish more reliable WC cut-points for different ethnic groups [11]. In the interim, the committee proposed that the WC cut-points as recommended by IDF are used until new evidence emerges [11]. The new JIS “harmonized” definition is expected to have the advantage of identifying a larger number of MetS cases in a population, compared to the other definitions.  Table 1 shows the summary of NCEP-ATP III, IDF, modified NCEP-ATP III, and JIS definitions for MetS. 

Robust evidence showed that individuals diagnosed with MetS using these definitions have a greater risk of significant clinical consequences, the two most prominent of which are the development of T2DM and CVD [12–15]. Individuals with MetS have a fivefold greater risk of developing T2DM [12], while a systematic review of 37 studies involving more than 170,000 patients has shown that MetS doubles the risk of cardiovascular events [15].

Irrespective of the diagnostic criteria used, epidemiological studies from various parts of the world have clearly demonstrated that MetS is an increasing global health problem, not only in the western societies, but also in the Asian populations [16, 17]. The clustering of CVD risk factors that characterizes MetS is a powerful driving force for the emerging CVD epidemic in Asia [17, 18]. The prevalence of MetS in East Asia was found to range from 8% to 13% in men and from 2% to 18% in women, depending on the population and definition used [17]. In Malaysia, epidemiological evidence describing prevalence of MetS is still scarce. Most local studies on MetS prevalence involved small sample size [19, 20], with the exception of a cross-sectional survey involving 4341 Malaysian adults (>18 years old) [21]. The survey showed that crude prevalence of MetS using WHO, NCEP-ATP III, IDF, and JIS definitions was 32.1%, 34.3%, 37.1%, and 42.5%, respectively [21]. More evidence is needed to describe the prevalence of MetS in Malaysia. This study aimed to compare the overall and age-adjusted prevalence of MetS in Malaysian adults using the NCEP-ATP III, IDF, and JIS definitions, identify the demographic factors associated with MetS, and determine the level of agreement between these definitions.

## 2. Methods

### 2.1. Study Design and Population

The Cardiovascular Risk Prevention Study (CRisPS) is an ongoing, prospective, community-based, cohort study involving Malaysian adults ≥30 years of age from 18 urban and 22 rural communities from the states of Selangor, Negeri Sembilan, Pahang, Kelantan, and Sabah. 

Subjects in CRisPS were selected in a four-stage sampling process: selecting the states and then “communities,” followed by households within them and finally individuals within households. This design enables identification of both individual and environmental determinants of health. 

### 2.2. Sampling Methods: State and Site Selection

**Figure 1:**
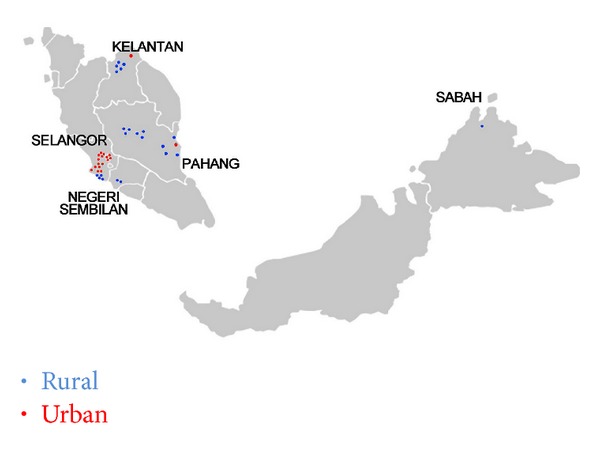
The Cardiovascular Risk Prevention Study (CRisPS) sites map.

The 5 states were pragmatically chosen to ensure adequate representation of the major ethnic groups in Malaysia. The main ethnic groups in Peninsular Malaysia are Malays, Chinese, and Indian, while the main ethnic groups in Sabah are the Kadazan-Dusun, Bajau, and Murut. For the purpose of this study, the Kadazan-Dusun, Bajau, Murut, and several other ethnic minorities were categorized as the indigenous groups. Selangor, Negeri Sembilan, and Pahang have a good mixture of Malay, Chinese, and Indian populations. Kelantan offers a predominant Malay population while Sabah represents the indigenous population.  Figure 1 shows the map of CRisP Study sites illustrating the distribution of the urban and rural communities across the 5 states.

Community was defined as groups of people residing in the same locality who have common characteristics such as culture, socioeconomics, and use of amenities, goods, and services. Urban and rural areas were defined according to the Malaysian Population and Housing Census (2000) [22]. Gazetted areas with a combined population of 10,000 or more were defined as urban areas, while areas with a population of less than 10,000 were classified as rural. 

Both urban and rural communities were selected with the aim of achieving within-community homogeneity in demographic and socioeconomic profiles. In view of the fact that this study was designed as a prospective cohort study, these communities were also selected based on the pragmatic requirement of optimizing the capacity of investigators to maintain long-term follow-up of participants and cooperation by community leaders. These factors were important to ensure continuity of data collection which is being scheduled every 3 years, for a period of 15 years.

### 2.3. Sampling Methods: Subjects Recruitment

A standardized method of recruitment was adopted, in which announcements and invitations were made through local community leaders. All household members aged ≥30 years residing in each locality were invited to attend a screening session at their local community centre through written invitation. Participants were given information regarding the purpose of the study and were requested to fast for at least 8 hours prior to the screening. A response rate of 60–70% was recorded for each site. At the screening centre, subjects were screened for eligibility. They were given an information leaflet regarding the study, and written informed consent was obtained. 

The study protocol was approved by the institutional ethics committee. 

### 2.4. Study Procedures

All interviewers and investigators were trained regarding the study procedures prior to the conduct of the study to minimize variability in the method of data collection. Standardized, interviewer-based questionnaires were used to collect information regarding age, gender, ethnic group, and educational attainment. 

Anthropometrical measures which included waist circumference (WC) and blood pressure (BP) were obtained. WC was measured to the nearest 0.1 cm by using nonstretchable measuring tape with the subjects standing in a relaxed position and arms at the side. The measurement was taken at the midpoint between the lower rib margin (12th rib) and the iliac crest. BP was measured twice 2 minutes apart on the right arm in sitting position, using Omron automatic digital blood pressure monitor (Omron HEM-757). Subjects were advised not to smoke, exercise, or eat in the last 30 minutes and not to climb stairs in the last 15–30 minutes and were made to rest for at least 5 minutes before the measurements were taken. Subjects were seated upright with his/her right arm supported at heart level. The average of the first and second measurements was used as the BP value for individual subjects. If the measurements differ by 5 mmHg of either systolic or diastolic readings, subsequent measurements were taken at 5–10 minutes apart. The process was repeated until two BP values which did not differ by more than 5 mmHg of either systolic or diastolic readings were obtained. The average of these two BP readings was used as the BP value for that particular subject.

Fasting venous blood samples were collected for plasma glucose and serum lipid profile (total cholesterol (TC), triglycerides (TG), low-density lipoprotein cholesterol (LDL-C), and high-density lipoprotein cholesterol [HDL-C]). TG, TC, HDL-C, and glucose levels were measured by enzymatic reference method on an automated analyzer (Cobas Integra 400 plus, Roche Diagnostic, Basel, Switzerland). 

### 2.5. Metabolic Syndrome Definitions

MetS was defined according to 3 sets of criteria proposed by the NCEP-ATP III 2001 [8], IDF 2005 [9], and JIS 2009 [11] as previously shown in Table 1. Instead of the modified NCEP-ATP III 2005 [10], the first version of NCEP-ATP III 2001 which applied Caucasian cut-points for WC was chosen to define MetS for the purpose of this paper as it allows better comparison with other western studies. Furthermore, the MetS criteria in the modified NCEP-ATP III 2005 are very similar to those in JIS 2009. Consequently, the analysis using these two definitions yielded similar results. Therefore, the modified NCEP-ATP III 2005 definition was excluded as it does not add further value to this paper.

### 2.6. Data Collection

The baseline data was collected from 2007 to 2011, and follow-up data is currently being collected every 3 years for a period of 15 years. The analytic sample presented in this paper consisted of 8,836 subjects who were recruited at baseline. 

### 2.7. Statistical Analysis

All data were entered and analyzed using STATA software version 11.1. Numerical variables were described with mean (±Standard Deviation (SD)). Categorical variables were described with frequency and percentage. Age-adjusted prevalence was computed using direct standardization method and presented with 95% CI. Multiple logistic regressions were used to determine the association between MetS according to the various definitions and the site of dwelling, age, gender, ethnicity, and educational attainment. Sensitivity, specificity, and level of agreement using Kappa index were determined for MetS diagnoses as defined by the NCEP-ATP III and JIS definitions, against the IDF definition. Significance level was set at *P* value of <0.05.

## 3. Results

A total of 8,836 adults (mean age 53.21 ± 10.6) participated in this study. The sociodemographic characteristics of the study subjects are shown in Table 2. It is noted that there were higher percentages of urban compared to rural populations and more females compared to male participants. With regard to ethnic group distributions, Malays comprised 72.5%, Chinese 12.3%, Indians 3.2%, and indigenous groups 12.0% of the study population. More than a third of the participants attained secondary education and about a fifth attained tertiary educational level. 

Table 3 shows the overall and age-adjusted prevalence of MetS according to the NCEP-ATP III, IDF, and JIS definitions by location, gender, ethnic group, and education attainment. JIS definition gave the highest overall prevalence (43.4%) compared to IDF (37.4%) and NCEP-ATP III (26.5%). There was significantly higher age-adjusted prevalence of MetS in the urban compared to the rural population according to the IDF (39.0% versus 36.3%, *P* = 0.002) and JIS (44.6% versus 42.5%, *P* = 0.018). In comparison to males, female participants had significantly higher age-adjusted prevalence according to the NCEP-ATP III (24.1% versus 28.9%, *P* < 0.001) and IDF (36.1% versus 39.3%, *P* = 0.001) but not significant according to the JIS definition (43.8% versus 43.9%, *P* = 0.777). The Chinese had the lowest age-adjusted prevalence according to the NCEP-ATP III definition (15.4%, *P* < 0.001), while the indigenous groups had the lowest age-adjusted prevalence by IDF (26.3%, *P* < 0.001) and JIS (32.5%, *P* < 0.001) definitions. Indians had significantly higher age-adjusted prevalence compared to other ethnic groups by all MetS definitions (30.1% by NCEP-ATP III, 50.8% by IDF, and 56.5% by JIS). According to the NCEP-ATP III, those with tertiary education had the lowest age-adjusted prevalence (19.5%, *P* < 0.001). On the contrary, those with no formal education had the lowest age-adjusted prevalence by IDF definition (32.3%, *P* = 0.03). 

Table 4 shows the association between MetS and the sociodemographic factors. The likelihood of having MetS amongst rural and urban populations was similar across all definitions. Female participants were found to be more likely to have MetS compared to males according to the NCEP-ATP III and IDF definitions but not by JIS definition. The likelihood of having MetS also increased with age where the odds ratio (OR) was highest in those aged ≥60 years. With regard to ethnicity, Chinese and indigenous groups were found to be less likely to have MetS compared to the Malays across all definitions. Indians were found to be more likely to have MetS compared to the Malays according to the IDF and JIS definitions. In terms of education attainment, subjects with tertiary education were less likely to have MetS according to the NCEP-ATP III definition compared to those with no formal education. According to the IDF and JIS definitions, subjects with primary education were more likely to have MetS compared to those with no formal education.

Table 5 shows the sensitivity and specificity of MetS as defined by the NCEP-ATP III and JIS against IDF definition as the reference criteria for diagnosis. The NCEP-ATP III definition was only able to identify 60.7% as having MetS out of the total IDF defined cases. There was a high false negative rate at 39.3%. The level of agreement as expressed by the Kappa index was 0.580. The JIS definition was successful in diagnosing 99.4% of those diagnosed with MetS according to the IDF definition. The false negative rate was very low at 0.6%. The level of agreement between the JIS and IDF definitions was very good with a Kappa index of 0.867.

## 4. Discussion

The CRisP Study reinforces the findings of a previous nationwide survey [21] that the overall prevalence of MetS among Malaysian adults was much higher compared with other Asian countries [17, 23]. This study found that the overall prevalence of MetS among Malaysian adults was 26.5%, 37.4%, and 43.4% according to the NCEP-ATP III, IDF, and JIS definitions, respectively. Comparison to other Asian countries can be made using the IDF definition (with similar WC cut-points), where the prevalence was much lower in Japan (11%) [24], Singapore (17.7%) [25], and Nepal (22.5%) [26]. Comparison to other populations is harder to make as different WC cut-points were used to define abdominal obesity. MetS prevalence in the Greek population, however, was higher at 43.4% by IDF and 45.7% by JIS [27]. Similarly, prevalence by NCEP ATP III in the United States (34.0%) [28] and Iran (34.7%) [29] was much higher compared to the finding in the CRisP Study. 

With regard to ethnicity, this study found that Malaysian Indians had significantly higher age-adjusted prevalence compared to other ethnic groups by all MetS definitions (30.1% by NCEP-ATP III, 50.8% by IDF, and 56.5% by JIS). Another study also found that the prevalence in Asian Indians was high at 35.8% by NCEP-ATP III and 39.5% by IDF criteria [30]. It is well recognized that Indians, in particular of South Asian origin, have an ethnic predisposition to abdominal obesity, glucose intolerance, hypertension, and dyslipidaemia, resulting in increased morbidity and mortality rates due to CVD [31]. On the contrary, this study shows that Malaysian Chinese had the lowest MetS prevalence by NCEP-ATP III, while the indigenous groups had the lowest prevalence by IDF and JIS definitions. Malaysian Chinese and indigenous groups were also found to be less likely to have MetS compared to the Malays according to all definitions. Variations in the MetS prevalence in different ethnic groups living in the same country had also been shown in several other studies [32]. This variation may be due to the environmental and genetic factors, which have long been recognized to play a pivotal role in the pathophysiology of MetS [33].

As expected, the JIS definition gave the highest overall prevalence of MetS compared to NCEP-ATP III and IDF definitions. The JIS definition offers an advantage of identifying a larger number of individuals with MetS due to the presence of other multiple risk factors, despite having WC of less than the recommended cut-points. In this study, the likelihood of males being identified to have MetS was similar to females according to JIS definition, but female participants were found to be more likely to have MetS compared to males according to the NCEP-ATP III and IDF definitions. This may be explained by the fact that a higher number of males were diagnosed to have MetS according to the JIS definition, despite having WC of less than 90 cm (hence not classified as having MetS by IDF criteria). The ORISCAV-LUX study involving 1349 European subjects found that MetS prevalence by JIS definition was significantly higher in men than in women, as were all components of MetS except abdominal obesity [34]. A prospective analysis of mortality in men found that these males were still at high risk of CVD mortality due to the presence of multiple risk factors despite the absence of abdominal obesity [35]. A recent review has suggested that JIS definition may better identify those at increased risk of CVD, while IDF definition may be more appropriate for the identification of those with insulin resistance and increased risk of T2DM [36].

In terms of location, this study shows that the likelihood of having MetS amongst the rural population was similar to their urban counterparts. The results are comparable to the NHMS III cohort study group whose data indicates that cardiovascular risk-factor clustering was similar in urban and rural populations [18]. The findings support the hypothesis that rapid economic development leads to urbanization of rural areas in Malaysia, resulting in the rural population adopting sedentary lifestyle, akin to their urban counterparts, and, therefore, have similar T2DM and CVD risks. 

Consistent with the findings from another study [34], the CRisP Study also shows the likelihood of having MetS increased with age, where the OR was highest in those aged ≥60 years. This is thought to be due to age-related rises of blood pressure and plasma glucose level [37]. Although Malaysia is still considered as a relatively young nation, its ageing population of ≥60 years is rising steadily from 5.7% in 1990 to 6.3% in 2000 and is expected to be 9.8% in 2020 [38]. The increasing number of elderly population with multiple risk factors and comorbidities is poised to become a major challenge to the Malaysian healthcare system in the near future [39].

With regard to determining the level of agreement between the three definitions, IDF was used as the reference criterion as it is the most widely accepted in Malaysia. This study found a very high level of agreement between the JIS and IDF definitions (Kappa index = 0.867). The finding is expected in view of similar reference range of the attributes including WC cut-points used in both definitions. On the contrary, there was a lower level of agreement (Kappa index = 0.580) between NCEP-ATP III and IDF. This may be attributed to the difference in the reference ranges and WC cut-points in the NCEP-ATP III definition. Similarly, the ORISCAV-LUX study showed a remarkable level of agreement between the JIS and IDF definitions (Kappa index = 0.93), and lower level of agreement was found between NCEP-ATP III and IDF definitions (Kappa index = 0.84) [34]. JIS should be recommended as the preferred diagnostic criterion as it shows a very high level of agreement with IDF and also has the advantage of identifying a larger number of individuals with MetS.

This study underlines the magnitude of cardiovascular risk factor clustering in the Malaysian population. The National Health and Morbidity Survey (2011) shows similar trend of escalating cardiovascular risk factor prevalence over the last two decades [40]. A higher MetS prevalence would translate into higher numbers with established diabetes, cardiovascular diseases, and events, leading to increased utilization of healthcare services, escalating health care costs, increased premature deaths, reduced productivity, and catastrophic economic implications [41]. This CVD epidemic has already begun in Malaysia where there is a trend towards a younger age at first myocardial infarction [42] and higher cardiovascular mortality than in developed countries [43].

MetS has now become a major public health threat and clinical problem. In response to the urgent needs to address this phenomenon, the Ministry of Health, Malaysia, launched the National Strategic Plan for the Non Communicable Disease (NSPNCD) in 2010, focusing on seven strategies which include prevention and health promotion, clinical interventions, partnership with patients, public engagement, research and surveillance, capacity building, and regulatory interventions [44]. However, when resources are limited, priorities should be given to the most cost-effective strategies which can produce swift changes [45]. At the public health level, more attention should be given to the modification of lifestyle of the general public through health promotion campaigns, multisectoral collaborations, and regulatory measures [45, 46]. Preventive strategies should begin early in childhood as 3.9% (0.3 million) of Malaysian children below 18 years were found to be obese [40]. Children who are obese have a greater likelihood of being obese in adulthood which would lead to the development of MetS, diabetes, and cardiovascular diseases [47]. Therefore, preventing obesity among young children is a vital strategy for reversing the epidemic [44]. The preschool and primary school years provide numerous opportunities to promote healthy eating and physical activity behaviors among children. Strategies which have been proven to be effective include integrating education on healthy eating, physical activity, and body image into classroom curriculum, increasing opportunities for physical activity throughout the school week, improving nutritional quality of the food supply in schools, engaging parents and promoting home activities, and supporting teachers to implement health promotion strategies [48]. Implementation of these interventions in Malaysian schools would require multisectoral collaborations between the education and health ministries. Regulatory measures should also include a requirement for the canteen operators to provide healthy food choices in Malaysian schools.

At the primary care level, individual patients with MetS need to be identified so that their multiple risk factors can be managed early to reduce complications [45]. Recent evidence from a middle income country has proven that treatment of multiple risk factors such as hypertension and dyslipidaemia had a much higher disability-adjusted life year (DALY) saved compared to enforced salt reduction in bread and tobacco cessation campaigns in mass media [49]. Concerted efforts should also be made to screen healthy individuals who are at risk of developing MetS. However, this attempt is often hampered by the shortage of multidisciplinary primary care team personnel in Malaysia [50]. Solutions therefore lie in enhancing the primary care delivery system and capacity building of primary care workforce [45, 50]. The recently announced proposal by the Malaysian government to integrate all public and private primary care workforces under a common network of care, supported by the national health financing scheme, offers a promise of better coordination, continuity, and quality of care, especially to individuals with multiple risk factors [51].

### 4.1. Limitation

This study has several limitations. In contrast to other cross-sectional epidemiological studies where multistage stratified random sampling designs were used, this ongoing prospective cohort study pragmatically selected the urban and rural communities based on the feasibility for long-term follow-up and cooperation by the community leaders. The findings of this study, however, are similar and comparable to the findings of a previous nationwide survey using a two-stage stratified random sampling design [21].

Malaysia's ethnic population comprises 53.3% Malays, 26.0% Chinese, and 7.7% Indians, and the remaining 13% are of other ethnic groups [22]. Although measures had been taken to ensure adequate representation of the country's population, Malays were overrepresented, while Chinese and Indian populations were underrepresented in this study. However, the large sample size of this study population should counterbalance any possible bias in the sampling. 

The possibility of determining which MetS definition better predicts adverse cardiovascular outcomes is also limited at this stage. The follow-up data of this ongoing prospective cohort study would be able to offer such findings in the future. 

## 5. Conclusion

In conclusion, regardless of the definitions used, the CRisP Study indicates high MetS prevalence among Malaysian adults, much higher than in other Asian countries. Findings from this study underscore the urgency to stem the rising tide of MetS prevalence and the almost inevitable epidemic of T2DM and CVD in Malaysia. Public health measures, as well as individual intervention in primary care, are crucial to reduce their risk of developing T2DM and CVD. The JIS definition offers the advantage of identifying a larger number of individuals with MetS and should therefore be recommended as the preferred diagnostic criterion.

## Figures and Tables

**Table 1:** Diagnostic criteria for metabolic syndrome.

Risk factors (RF)	NCEP-ATP III (2001)	IDF (2005)	Modified NCEP-ATP III (2005)	JIS (2009)
Waist circumference (WC)	M ≥ 102 cm F ≥ 88 cm (Caucasian cut-points)	M ≥ 90 cm F ≥ 80 cm (South Asian cut-points)	M ≥ 90 cm F ≥ 80 cm (Asian cut-points)	M ≥ 90 cm F ≥ 80 cm (South Asian cut-points)
Blood pressure (BP)	Systolic BP ≥ 130 and/or diastolic BP ≥ 85 mmHg	Systolic BP ≥ 130 and/or diastolic BP ≥ 85 mmHg or on treatment for HPT	Systolic BP ≥ 130 and/or diastolic BP ≥ 85 mmHg or on treatment for HPT	Systolic BP ≥ 130 and/or diastolic BP ≥ 85 mmHg or on treatment for HPT
Fasting plasma glucose (FPG)	≥6.1 mmol/L	≥5.6 mmol/L or previously diagnosed T2DM	≥5.6 mmol/L or on treatment for elevated glucose	≥5.6 mmol/L or on treatment for elevated glucose
Triglycerides (TG)	≥1.7 mmol/L	≥1.7 mmol/L or on treatment for TG	≥1.7 mmol/L or on treatment for TG	≥1.7 mmol/L or on treatment for TG
HDL-C	M < 1.03 mmol/L F < 1.29 mmol/L	M < 1.03 mmol/L F < 1.29 mmol/L or on treatment for HDL-C	M < 1.03 mmol/L F < 1.29 mmol/L or on treatment for HDL-C	M < 1.0 mmol/L F < 1.3 mmol/L or on treatment for HDL-C

Metabolic syndrome definitions	At least 3 RF	WC + 2 or more RF	At least 3 RF	At least 3 RF

RF: risk factors.

**Table 2:** Demographic characteristics of the study subjects.

Demographic characteristics	
All subjects, *n* (%)	8836 (100)
Mean age in years (±SD)	53.21 (±10.6)
Location, *n* (%)	
Urban	4757 (53.8)
Rural	4079 (46.2)
Gender, *n* (%)	
Male	3766 (42.6)
Female	5070 (57.4)
Ethnic group, *n* (%)	
Malays	6408 (72.5)
Chinese	1085 (12.3)
Indians	280 (3.2)
Indigenous groups	1063 (12.0)
Education attainment, *n* (%)*	
No formal education	1227 (14.7)
Primary	2182 (26.1)
Secondary	3236 (38.7)
Tertiary	1715 (20.5)

*Number not equal to *n* = 8836 due to missing data (*n* = 8360).

**Table 3:** The overall and age-adjusted prevalence of metabolic syndrome according to the NCEP-ATP III, IDF, and JIS definitions by location, gender, ethnic group, and education attainment.

Demographic characteristics	Total number of subjects	NCEP-ATP III (2001)	IDF (2005)	JIS (2009)
		Prevalence (95% confidence interval)		
Overall	8836	26.5 (25.6–27.4)	37.4 (36.3–38.4)	43.4 (42.3–44.4)
*P* value		<0.001*		
Location				
Urban	4757	25.0 (23.8–26.2)	39.0 (37.7–40.4)	44.6 (43.2–46.0)
Rural	4079	28.5 (27.1–29.9)	36.3 (34.8–37.7)	42.5 (41.0–44.0)
*P* value		0.001*	0.002*	0.018*
Gender				
Male	3766	24.1 (22.7–25.4)	36.1 (34.6–37.7)	43.8 (42.3–45.4)
Female	5070	28.9 (27.6–30.1)	39.3 (38.0–40.6)	43.9 (42.6–45.2)
*P* value		<0.001*	0.001*	0.777
Ethnic group				
Malays	6408	29.1 (28.0–30.2)	40.2 (39.1–41.4)	46.0 (44.8–47.1)
Chinese	1085	15.4 (13.3–17.6)	27.9 (25.3–30.5)	33.4 (30.7–36.0)
Indians	280	30.1 (25.3–34.9)	50.8 (45.6–56.0)	56.5 (51.4–61.7)
Indigenous groups	1063	20.7 (18.5–23.2)	26.3 (23.6–29.1)	32.5 (29.6–35.4)
*P* value		<0.001*	<0.001*	<0.001*
Education attainment				
No formal education	1227	25.4 (22.6–28.2)	32.3 (29.2–35.4)	39.8 (36.6–43.0)
Primary	2182	29.4 (27.4–31.3)	38.5 (36.4–40.5)	44.4 (42.3–46.6)
Secondary	3236	27.1 (25.5–28.8)	39.4 (37.7–41.2)	44.9 (43.1–46.7)
Tertiary	1715	19.5 (17.4–21.6)	35.3 (32.8–37.7)	40.8 (38.3–43.4)
*P* value		<0.001*	0.03*	0.362

*P value < 0.05.

**Table 4:** Factors associated with metabolic syndrome according to the ATP III, IDF, and JIS definitions among Malaysian adults ≥30 years of age.

Metabolic syndrome definitions	NCEP-ATP III (2001)		IDF (2005)		JIS (2009)	
	Odds ratio (95% CI)	*P* value	Odds ratio (95% CI)	*P* value	Odds ratio (95% CI)	*P* value
Location						
Urban	1.00		1.00		1.00	
Rural	1.06 (0.94–1.20)	0.326	0.87 (0.79–1.02)	0.086	0.89 (0.80–1.00)	0.058
Gender						
Male	1.00		1.00		1.00	
Female	1.29 (1.17–1.44)	<0.001*	1.19 (1.09–1.31)	<0.001*	1.02 (0.93–1.12)	0.616
Age (years)						
30–39	1.00		1.00		1.00	
40–49	1.39 (1.13–1.73)	<0.001*	1.63 (1.35–1.97)	<0.001*	1.56 (1.30–1.87)	<0.001*
50–59	2.26 (1.83–2.79)	<0.001*	2.72 (2.25–3.27)	<0.001*	2.72 (2.27–3.25)	<0.001*
≥60	2.34 (1.87–2.92)	<0.001*	2.67 (2.18–3.26)	<0.001*	3.08 (3.54–3.70)	<0.001*
Ethnicity						
Malay	1.00		1.00		1.00	
Chinese	0.44 (0.36–0.53)	<0.001*	0.53 (0.45–0.62)	<0.001*	0.56 (0.49–0.66)	<0.001*
Indian	1.02 (0.77–1.37)	0.867	1.48 (1.14–1.92)	0.003*	1.54 (1.18–1.99)	<0.001*
Indigenous groups	0.60 (0.50–0.72)	<0.001*	0.56 (0.47–0.66)	<0.001*	0.63 (0.54–0.73)	<0.001*
Education level						
No formal education	1.00		1.00		1.00	
Primary	1.16 (0.99–1.37)	0.067	1.34 (1.15–1.58)	<0.001*	1.18 (1.01–1.37)	0.029*
Secondary	1.05 (0.89–1.25)	0.544	1.25 (1.06–1.48)	0.009*	1.12 (0.96–1.31)	0.147
Tertiary	0.77 (0.63–0.95)	0.012*	1.04 (0.86–1.27)	0.673	0.92 (0.76–1.10)	0.350

*P value < 0.05.

**Table 5:** Sensitivity, specificity, and level of agreement for metabolic syndrome as defined by the NCEP-ATP III and JIS definitions against the IDF definition.

Definitions	IDF					
	No MetS	Yes MetS	Sensitivity	Specificity	Kappa index	*P* value
NCEP-ATP III						
No MetS	5194 (93.9%)	1299 (39.3%)	60.7%	93.9%	0.580	<0.001*
Yes MetS	337 (6.1%)	2006 (60.7%)				
JIS						
No MetS	4981 (90.1%)	19 (0.6%)	99.4%	90.1%	0.867	<0.001*
Yes MetS	550 (9.9%)	3286 (99.4%)				

*P value < 0.05.
